# Gene silencing in HIV-1 latency by polycomb repressive group

**DOI:** 10.1186/1743-422X-8-179

**Published:** 2011-04-18

**Authors:** Hyeon Guk Kim, Kyung-Chang Kim, Tae-Young Roh, Jihwan Park, Kyung-Min Jung, Joo-Shil Lee, Sang-Yun Choi, Sung Soon Kim, Byeong-Sun Choi

**Affiliations:** 1 Division of AIDS, Center for Immunology and Pathology, Korea National Institute of Health, Chung-buk, Republic of Korea; 2 Division of Molecular and Life Sciences; 3 Division of Integrative Biosciences and Biotechnology, Pohang University of Science and Technology (POSTECH), Gyeongbuk, Republic of Korea; 4 School of Life Science and Biotechnology, Korea University, Seoul, Republic of Korea

## Abstract

**Background:**

The persistence of latently Human immunodeficiency virus-1 (HIV-1) infected cellular reservoirs in resting CD4^+ ^T cells is a major obstacle to HIV-1 eradication. The detailed mechanism of HIV-1 latency remains unclear. We investigated histones and their post-translational modification associated with HIV-1 latency in novel HIV-1 latently infected cell lines established previously, NCHA cells.

**Methods:**

To examine histones and their modification linked with HIV-1 latency, the expression profiles for core histone proteins and histone deacetylases (HDACs) in NCHA cells were characterized by RT-PCR, ELISA, and western blot. The levels of histone acetylation and methylation at histone H3 Lys^9 ^(H3K9) and Lys^27 ^(H3K27) in HIV-1 latently infected cells were analyzed by western blot and chromatin immunoprecipitation-sequencing (ChIP-seq).

**Results:**

The expression levels for four core histone proteins (H2A, H2B, H3 and H4) and HDACs (HDAC1-8) in NCHA cells were not significantly different from those in their parental cells. Histone H3K9 and H3K27 acetylations in NCHA cells showed no difference in parental and NCHA cells, whereas the levels of di- and tri-methylation were increased in NCHA cells. The expression of EED which is a component of polycomb repressive complex 2 (PRC2), and BMI1 and RING2 which are constituents of PRC1, were upregulated in NCHA cells. In addition, more ubiquitylation at histone H2A was detected in NCHA cells.

**Conclusions:**

Our results suggest that tri-methylation of histone H3K27 and H2A ubiquitylation via polycomb group protein may play a crucial role in epigenetic silencing accounting for HIV-1 latency in NCHA cells.

## Background

HIV-1 can evade the immune responses of host cells and establish latent infection among HIV-infected patients, which leads to AIDS despite treatment referred to a highly active antiretroviral therapy (HAART). Latently infected memory T cells, which are generally established within days of the initial infection, are very rare (~1 million) in a patient and have a long half-life of over 44 months on average. The complete elimination of HIV reservoirs seems to take over 60 years under the HAART regimen [1].

In some HIV-1 latency studies, phosphorylation of NF-κB p65 activates the long terminal repeat (LTR) of HIV-1 provirus in latent cells directly, which in turn drives HIV-1 transcription. Phosphorylation of p65 at ser^276 ^enhances the recruitment of coactivator p300/HAT, leading to increased transcriptional activation in NF-κB dependent genes by the acetylation of histones [2,3]. NF-κB p50/HDAC complexes which constitutively bind to the HIV-1 LTR and maintain HIV latency can be depleted from the latent HIV promoter upon TNF-α induction [4]. Even if some researchers have reported that HDACs play a critical role in HIV-1 latency, molecular mechanisms involved in HIV-1 latency should be further explored for complete eradication of HIV.

The eukaryotic genome is organized into the highly complex nucleoprotein structure of chromatin. Epigenetic studies show that this chromatin structure can be modified by DNA methylation, covalent histone modifications, and nucleosome remodeling. In particular, histone proteins consisting of the nucleosome core are subject to modifications such as methylation, acetylation, ubiquitylation, sumoylation, and phosphorylation on specific residues [5]. Posttranslational modifications of core histones change their interactions with DNA and thus involve in diverse biological processes such as DNA repair, chromosome condensation, and gene regulation. Specifically, histone H3 Lys^4 ^tri-methylation (H3K4me3) is mostly enriched at active gene promoters, whereas histone H3K9 and H3K27 di-/tri-methylation (H3K9me2/me3 and H3K27me2/me3) are highly enriched at inactive loci in mammalian cells [6].

Recent cancer studies show that broad alteration of gene activation or silencing by epigenetic alterations can lead to cancer development and carcinogenesis [7]. Elevated expression levels of some histone methyltransferase such as EZH2 and G9a are associated with breast and liver cancers [8,9]. It has been suggested that HIV-1 latency is strongly associated with chromatin silencing and that novel drug development can be based on the chromatin modification like acetylation/deacetylation and methylation as new targets for HIV-1 therapy [1,10,11]. Recently, it was reported that G9a, one of histone methyltransferase, and p-TEFb contribute to the maintenance and establishment of HIV-1 latency [12,13]. In our previous study, HIV-1 latently infected cells also showed distinctly different patterns in histone H3K9 and H3K27 modification from those in their HIV-uninfected parental cells (unpublished data). Based on these findings, we tried to figure out the detailed mechanisms of HIV-1 silencing in the cellular reservoirs for curing HIV-1 infection from AIDS patients in the future.

## Materials and methods

### Cell lines

Reference cell lines, A3.01 cells and ACH2 cells, were obtained from the National Institutes of Health AIDS Research and Reference Reagent Program. Latently infected A3.01-derived cell lines, NCHA1 and NCHA2, were established from our laboratory [14]. Cells were cultured in RPMI 1640 culture medium supplemented with 10% fetal bovine serum (FBS), 5% penicillin/streptomycin, and 2mM glutamine at 37°C in humidified 5% CO2 incubator.

### RNA extraction and RT-PCR

TRIZol reagent (Invitrogen, USA) was used for isolation of RNA according to the manufacturer's protocol. Extracted RNA was stored at -20°C prior to use for RT-PCR. Total RNA (2ug) collected from each cell line was reverse transcribed to cDNA with Superscript II Reverse Transcriptase (Invitrogen) and an oligo(dT) primer.

### Preparation of nuclear extracts

Nuclear extracts were prepared with Nuclear Extraction Kit (Panomics, USA) and protein concentration of these nuclear extracts was measured with Protein DC assay Kit (Bio-Rad, USA) according to the manufacturer's protocol. Nuclear extracts were applied to ELISA assay immediately and the rest were stored at -80°C prior to use.

### ELISA assay for histone acetylation

Acetylation levels of four core histones were measured with Pathscan Acetyl Histone H2A, H2B, H3 and H4 Sandwich ELISA Kit (Cell Signaling, USA) according to the manufacturer's instruction.

### Western blot analysis

To identify HDAC proteins, total cellular protein extracts were prepared in ice-cold RIPA buffer (50 mM Tris pH 7.5, 0.5% deoxycholate, 0.5% NP-40, 0.5% SDS, and 100 mM NaCl) supplemented with fresh Complete™ protease inhibitor cocktail (Roche, Swiss). Protein extracts were separated by SDS-PAGE, transferred to an Immobilon™-P membrane (Millipore, USA), and probed with specific antibodies (H2A~ H4, HDACs and EED, Abcam, USA; ubiquitylated H2A, Millipore, USA). GAPDH (#2118, cell signaling, USA) and Lamin B (sc-20682, Santa Cruz, USA) were used as protein loading control. After incubation with the HRP-conjugated secondary antibody, proteins were visualized using West Pico chemiluminescence substrate (Pierce, USA) according to the manufacture's protocol.

### Immunoprecipitation

Immunoprecipitation was performed using IP Kit (Active motif, USA) with mouse anti-BMI1 (ab14389, Abcam, USA). The interaction of RING2 with BMI1 was examined with western blot using rabbit anti-RING2 (ab28629, Abcam, USA).

### Chromatin immunoprecipitation (ChIP)

Chromatin from uninfected and latently infected CD4+ T cells was digested with micrococcal nuclease into nucleosome-size unit. ChIP was performed using antibodies to histone H3K4me3 (ab8580, Abcam, USA), H3K9me3 (ab8898, Abcam, USA), H3K27me3 (07-449, Millipore, USA), and H3K9ac (ab4441, Abcam, USA). Following treatment of proteinase K, the DNA fragments were purified by phenol/chloroform extraction and ethanol precipitation.

### Genomic DNA library construction for ChIP-Seq and data analysis

The DNA sequencing library preparation and sequencing on the Genome Analyzer II were performed according to the manufacturer's instruction (Illumina, USA). Briefly, the ChIPed DNA was polished by blunting and 'A'-base addition followed by adapter ligation. After sequencing, the GA pipeline 1.4 software provided by Illumina was used for image analysis, base calling, and sequence alignment. The uniquely aligned sequence tags to the human genome (hg18/NCBI build 36) were used in further analysis. The sequence tags were counted in promoters (transcription start site ± 1kb), genebodies (from transcription start site + 1kb to transcription end site), and intergenic regions. To analyze the enrichment patterns of histone modifications on HIV-1 provirus, we aligned the sequencing tags to the HIV-1 genome and counted tags in a non-0verlapping 200bp window.

## Results

### Histone and HDAC levels in NCHA cells were not significantly different

**Figure 1:**
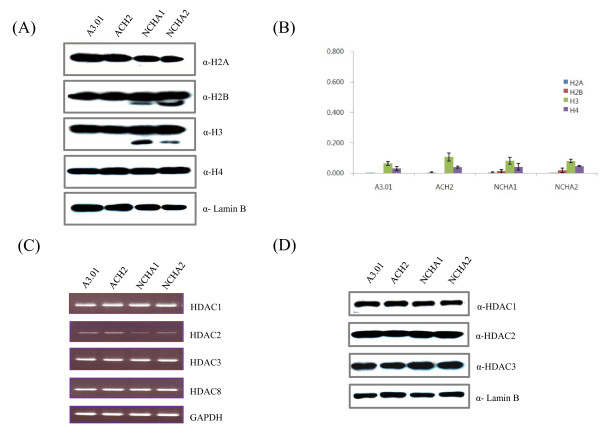
**Histone and HDAC profiles in HIV-1 latently infected A3.01derived cells**. A) Expression levels of four core histone proteins were investigated by western blot. Thirty ug of nuclear extract were loaded and immunoblotted using antibodies specific to H2A, H2B, H3 and H4. Anti-laminB was used as a loading control. B) The acetylation levels of four core histone proteins were quantified using the H2A-, H2B-, H3- and H4 -PathScan acetylated histone sandwich ELISA kit. Error bar represent the S.D. from the mean. C) mRNA levels of HDACs in each cell line were measured with reverse transcriptase PCR (RT-PCR). GAPDH was used as a control. D) Protein levels of HDAC1, HDAC2 and HDAC3 were investigated by western blot using 30 ug of nuclear extract. Anti-laminB was used as a loading control.

The expression and acetylation levels of four core histones (H2A, H2B, H3 and H4) in NCHA cells did not exhibit significant differences as shown in Figrue 1A and 1B, respectively. The mRNA levels for HDACs in Class I (HDAC1, 2, 3, and 8) were measured by RT-PCR analysis shown in Figure 1C. Both Class I and Class II HDACs (HDAC4, 6, and 7; data not shown) did not show any significant difference in parental and latently HIV-1 infected cells. The protein levels of HDAC1 - 3 in ACH2 and NCHA cells were relatively similar to those observed in their parent cells (Figure 1D; data not shown for HDAC4, 6, 7 and 8).

### Histone methylations were highly enriched in NCHA cells

**Figure 2:**
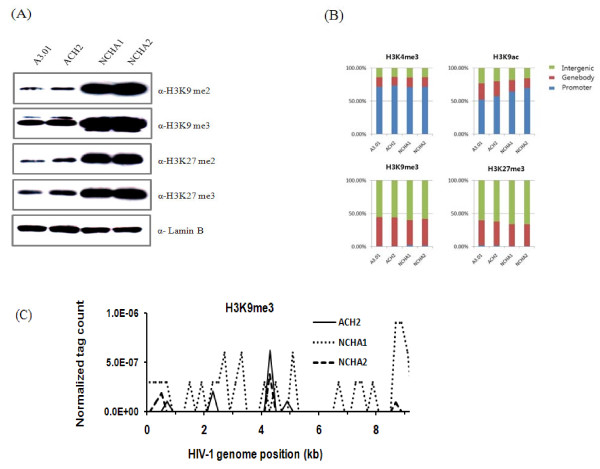
**Gene silencing by polycomb group-proteins in NCHA cells for HIV-1 latency**. A) Methylation levels of histone H3 on the specific lysine residues were measured by western blot. In each lane, 30 ug of nuclear extract were used and the antibodies were indicated on the right. Anti-laminB was used as a loading control. B) Global distributions of histone modifications were examined by ChIP-Seq. Promoters were the regions of 1 kb upstream and downstream of the transcription start site, and gene body was defined as the length from 1 kb downstream of the transcription start site to transcription end site and the remaining regions were intergenic regions. C) The H3K9me3 profile was shown on HIV-1 provirus genome. The X axis represents the HIV-1 genomic position and the Y axis indicates the normalized tag count which is calculated by total tag numbers detected in a 1 kb window divided by total sequenced tag number.

As shown in Figure 2A, the levels of di- and trimethylation of histone H3K9 and H3K27 were dramatically elevated in NCHA cells except ACH2 cells. To determine whether the elevation of H3K9me3 and H3K27me3 levels were biased in specific genomic regions, the genome-wide distribution of modifications were examined using all RefSeq genes (Figure 2B). H3K4me3 and H3K9ac were highly enriched in promoter regions, whereas H3K9me3 and H3K27me3 were distributed over the gene bodies and intergenic regions. However, the overall population was not significantly changed in ACH2 and NCHA cells compared with those of the A3.01 cells. The histone modification patterns of the HIV-1 proviral genomes showed that H3K9me3 was enriched in latently infected cell lines (ACH2, NCHA1, and NCHA2) shown in Figure 2C but H3K27me3 was not (data not shown).

### Polycomb group might induce HIV-1 gene silencing

**Figure 3:**
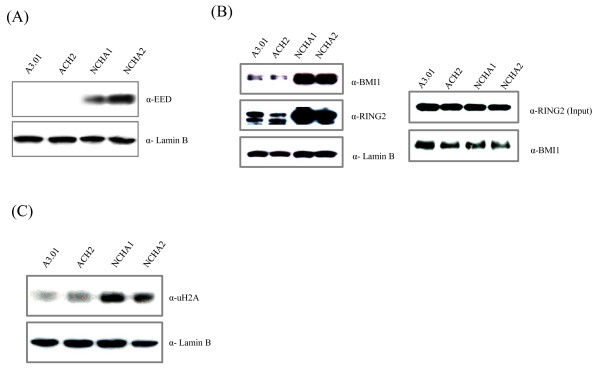
**Induction of HIV-1 gene silencing by polycomb group proteins**. A) Expression levels of EED, one of PRC2 components, were measured by western blot. Thirty ug of nuclear extract were also used. The antibodies were indicated on the right. B) The expression levels of polycomb group proteins, BMI1 and RING2, were examined by western blot. The right panel showed coimmunoprecipitation experiment, immunoprecipitated with RING2 antibody and blotted with BMI1 antibody. C) Ubiquitylation levels of all cell lines were investigated by western blot. Using 30 ug of nuclear extract and antibody against ubiqutylated histone H2A. Anti-laminB was used as a loading control.

To understand HIV-1 latency related with gene silencing by polycomb group protein complex, we examined the expression levels of polycomb group proteins in NCHA cells. Firstly, the expression level of EED, which is part of PRC2 that recognizes histone H3K27 and/or H3K9 residues for histone methylation, was investigated by western blot using an antibody specific to EED. As shown in Figure 3A, the expression of EED was upregulated in NCHA cells. Secondly, the expression levels of BMI1 and RING2, which are components of PRC1 that recognizes and binds to PRC2, were also increased in the nuclear fraction of NCHA cells compared with their parent cells and the interaction between these two proteins was confirmed by coimmunoprecipitation (Figure 3B). Finally, ubiquitylation levels of NCHA cells on histone H2A were also enriched compared with the parent cells (Figure 3C). These results suggest that PRC-mediated repressive H3K27 methylation and H2A ubiquitylation may contribute to HIV-1 gene silencing in NCHA cells.

## Discussion

Current antiretroviral therapies, including HAART, do not completely eradicate latent reservoirs of HIV-1 from patients, even though they suppress new HIV-1 replication effectively. The best way to eliminate this reservoir completely is to reactivate HIV-1 latently infected cells by inducing the expression of HIV-1 provirus. Many studies have reported potential antireservoir therapies to reactivate HIV-1 latently infected cells using HDAC inhibitors [15-17], interleukin-7 (IL-7) [18,19], hexamethyl-bisacetamide (HMBA) [20], and prostratin [21]. Eukaryotic genomes are organized into the chromatin structure with repeated nucleosomes, which consist of ~146 base pairs of DNA wrapped around an octamer of core histones (H3, H4, H2A, and H2B) [22]. Chromatin structure can be varied by DNA methylation and posttranslational histone modifications. These modifications are important for the regulation of cellular transcription, DNA replication, and repair [5,6]. Although some reports suggest that the chromatin structure at the HIV-1 integration site and LTR region plays a major role in HIV-1 postintegration latency [23,24], the molecular linkage between HIV-1 latency and epigenetic control, especially histone modifications, is not fully understood. In this study, to identify the epigenetic relevance to HIV-1 latency, we investigated core histones and histone modification factors such as HDACs, which were reported as critical factors in the maintenance of HIV-1 latency. As shown in Figure 1, the expression levels of four core histones and HDACs did not exhibit significantly differences in ACH2 and NCHA cells comparing to those in their parent cells. These results suggest that the protein amount of histones and HDACs in NCHA cells may not be associated with HIV-1 latency even though several studies have suggested HDACs-related HIV-1 latency models [3,4].

Interestingly, H3K9me2/me3 and H3K27me2/me3 were highly enriched in HIV-latent cells. To determine whether the enrichment of H3K9me3 and H3K27me3 in NCHA cells was specific to certain genomic regions, the distribution of histone methylations was examined in a genome-wide level. H3K4me3 and H3K9ac showed high enrichment in promoter regions, whereas H3K9me3 and H3K27me3 were spread out over gene bodies and intergenic regions. However, these patterns were not significantly changed in ACH2 and NCHA cells compared with those of A3.01 cells. To identify the chromatin states of HIV-1 proviral genome, we analyzed the histone methylation patterns at H3K9 and H3K27. High enrichment of H3K9me3 was observed in HIV-1 infected latent cells, whereas there was no enrichment H3K27me3. Our results show the possibility that the HIV-1 genome silencing may be mainly caused by H3K9me3-mediated process and the host cell genomes, especially in NCHA cells, can be regulated by both H3K9me3 and H3K27me3. These gene repression through histone methylations were consistent with the previous report showing that histone H3K27 trimethylation correlated with gene silencing in human T cells [25].

Polycomb group (PcG)-proteins mediated gene silencing is mainly established by histone H3K27 methylation. PcG proteins, which were first identified in *Drosophila melanogaster*, are required for the maintenance of HOX gene cluster repression during development. They induce transcriptional gene silencing in higher eukaryote and act as a global chromatin regulator [26-28]. PcG proteins consist of two different complexes, which are termed polycomb repressive complex 1 (PRC1) and 2 (PRC2) [27,28]. PRC1 is formed by over 10 different proteins including BMI1 and RING1A/B and ubiquitylates histone H2A by the E3 ubiquitin ligase activity of RING1A/B for silencing. PRC2 is a smaller complex that comprises three main proteins (EZH2, EED and SUZ12) and catalyzes histone H3K27me3 [29,30]. Recently, it was also reported that carboxy terminal domain of EED plays important role in binding to tri-methyl lysine residue of histone tails and is indispensable for the binding of PRC2 complex to repressive marks [31]. According to the known PcG-mediated silencing mechanism, PRC2 recruits PRC1 to target genes via the binding of PRC1 to histone H3K27me3. Subsequently, PRC1 catalyzes the ubiquitylation of H2A and may lead to specific gene silencing. Recently, it was reported that BMI1 play an important role in the maintenance of HSV-1 latency [32]. To elucidate the HIV-1 latency related to H3K27me3 in more detail, we examined the expression levels of EED, BMI1 and RING2 all of which were enriched in NCHA cells and the interaction of BMI1 and RING2 was identified. Also, the ubiquitylation levels of histone H2A in NCHA cells were increased compared with the parent cells. These results suggest that PRC-mediated repressive H3K27 methylation, H3K27me3, and H2A ubiquitylation may contribute to HIV-1 gene silencing in NCHA cells.

## Conclusion

Newly established NCHA cells harboring latent HIV-1 showed the enrichment of histone H3K27 methylation, histone H2A ubiquitylation, and PcG proteins expression. Therefore, our result demonstrates that tri-methylation of H3K27 and H2A ubiquitylation via polycomb repressive complexes should be involved in HIV-1 latency and contribute to epigenetic gene silencing.

## Competing interests

The authors declare that they have no competing interests.

## Authors' contributions

BSC and HGK conceived the project and designed experiments. HGK and KCK carried out research, wrote the manuscript and prepared the figures. KMJ, TYR and JP carried out CHIP and CHIP-seq. JSL, SYC and SSK helped to draft the manuscript. All authors approved the final manuscript.
